# Clinical efficacy and pharmacological mechanism of Er Zhi Tian Gui formula in enhancing IVF-ET outcomes for diminished ovarian reserve

**DOI:** 10.3389/fphar.2025.1552581

**Published:** 2025-03-21

**Authors:** Wen Chen, Fang Lian, Jingyan Song, Chen Yu, Shengyong Ding, Haicui Wu

**Affiliations:** ^1^ First School of Clinical Medicine, Shandong University of Traditional Chinese Medicine, Jinan, China; ^2^ Department of Reproduction and Genetics, Affiliated Hospital of Shandong University of Traditional Chinese Medicine, Jinan, China; ^3^ Department of Pharmacy, Affiliated Hospital of Shandong University of Traditional Chinese Medicine, Jinan, China

**Keywords:** diminished ovarian reserve, Er Zhi Tian Gui formula, reproductive Health, traditional Chinese Medicine, oocyte quality, granulosa cell apoptosis

## Abstract

**Objective:**

To investigate the efficacy of the Er Zhi Tian Gui Formula (EZTGF) in diminished ovarian reserve (DOR) patients undergoing *in vitro* fertilization-embryo transfer (IVF-ET); to examine the pharmacological mechanism of EZTGF in inhibiting ovarian granulosa cell (OGC) apoptosis.

**Methods:**

A total of 120 DOR patients undergoing IVF-ET in the Affiliated Hospital of Shandong University of TCM from March to December 2021 were randomly assigned to the EZTGF (n = 60) or Placebo (n = 60) groups. All participants followed a gonadotropin-releasing hormone antagonist protocol for controlled ovarian stimulation, with interventions starting on days 2–3 of the preceding menstrual cycle and continuing until the trigger day. IVF-ET outcomes measured included the number of oocytes retrieved, embryo quality, and pregnancy rates. NEAT1, miR-10b-3p, and FOXO3a OGCs expression was analyzed via qRT-PCR. Mechanistic studies were conducted using KGN cells, with dual-luciferase reporter assays confirming regulatory relationship between NEAT1, miR-10b-3p, and FOXO3a.

**Results:**

A total of 116 patients completed the trial (58 in each group). The EZTGF group showed a significant increase in both the number (especially at the cleavage stage) and rate of high-quality embryos, compared with the Placebo group (P < 0.05). No statistically significant differences in pregnancy outcomes were observed (P > 0.05). Cellular experiments showed that miR-10b-3p inhibits proliferation and promotes apoptosis in oocyte granulosa cells (OGCs). Dual luciferase reporter gene assay confirms target-regulatory relationship of NEAT1 with miR-10b-3p, miR-10b-3p and FOXO3a. EZTGF treatment partially reversed the low expression of NEAT1 and FOXO3a, and the high expression of miR-10b-3p in OGCs from DOR patients.

**Conclusion:**

Treatment with EZTGF enhances embryo quality in women with DOR undergoing IVF-ET, which can be partially attributed to modulation of the NEAT1/miR-10b-3p/FOXO3a pathway. Trial registration: ChiCTR2100052522.

## Graphical Abstract

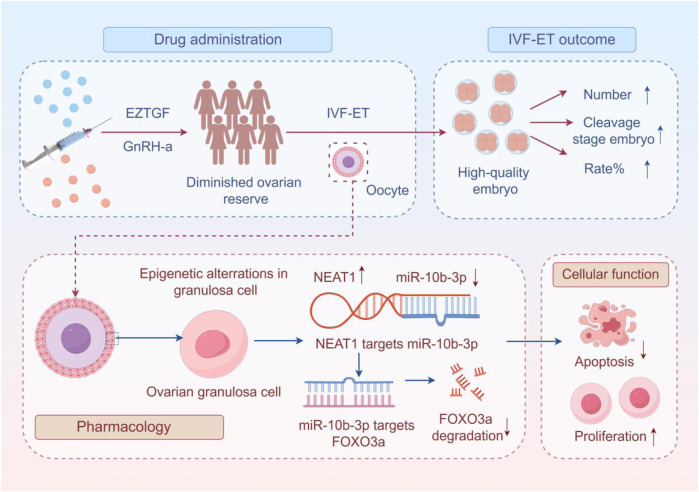

## 1 Introduction

In recent years, the global incidence of infertility has witnessed a significant increase, driven by factors such as environmental pollution, shifting fertility attitudes, and escalating societal pressures (Dehkordi et al., 2025; Liu et al., 2025). This trend has elevated infertility to a critical medical and sociological challenge with far-reaching implications worldwide (Bindeman et al., 2025). Diminished ovarian reserve (DOR), characterized by a decline in both the quantity and quality of oocytes, represents one of the significant contributing factors to infertility (Inhorn and Patrizio, 2015). For DOR patients with fertility aspirations, early access to fertility counseling and intervention is crucial. *In vitro* fertilization-embryo transfer (IVF-ET) has addressed some infertility issues (Christodoulaki et al., 2021), patients with DOR still experience a limited number of oocytes retrieved and good quality embryos (Levi et al., 2001). Western medicine lacks a universally effective ovulation induction regimen, and while gonadotropin-releasing hormone (GnRH) antagonist protocols are widely used due to their advantages—such as lower gonadotropin dosage, cost-effectiveness, good patient compliance, and low medical risk—their clinical efficacy remains limited (Depalo et al., 2012). Given the irreversible nature of ovarian reserve depletion, extensive research has been conducted by medical scientists to explore therapeutic interventions for DOR (Agarwal et al., 2018; Hu et al., 2023; Martirosyan et al., 2023). Currently, traditional Chinese medicine (TCM) and novel pharmacological interventions have been investigated as adjunctive approaches to enhance oocyte quality. These combined strategies aim to address the multifaceted challenges associated with DOR and improve reproductive outcomes.

The Er Zhi Tian Gui formula (二至天癸方, EZTGF) is a traditional Chinese herbal remedy designed to nourish kidney yin and support reproductive function. In conjunction with an ovulation promotion regimen, EZTGF has been shown to improve the quality of embryos fertilized *in vitro*, particularly in DOR patients with kidney qi and yin deficiency (Liu et al., 2023). It was developed by Professor Fang Lian, a National Famous Chinese Medicine Practitioner, based on the classical TCM that “the kidney is the master of reproduction,” along with over 40 years of clinical experience. This formula has been used for many years as a proprietary preparation at the Affiliated Hospital of Shandong University of TCM. It is typically administeblue alongside ovarian stimulation protocols to enhance embryo quality and improve pregnancy outcomes in patients with DOR and advanced age, but clinical studies in patients with DOR have not been reported. Therefore, we designed a randomised, double-blind, placebo-controlled clinical trial with the aim of investigating the clinical efficacy and safety of EZTGF in the treatment of DOR.

Previous study found that EZTGF could inhibit ovarian granulosa cell (OGC) apoptosis and improve clinical pregnancy rate in IVF-ET patients with renal qi deficiency, but the intrinsic mechanism remains unclear (Lian and Xiang, 2017). OGCs, a fundamental component of ovarian tissue, are crucial for maintaining normal ovarian functions, such as hormone synthesis and ovulation (Martinez et al., 2023). Nuclear paraspeckle assembly transcript 1 (NEAT1) has been identified as a key long non-coding RNA (lncRNA) that is significantly underexpressed in patients with DOR (Dong et al., 2022). In previous *in vitro* experiments, NEAT1 overexpression was shown to inhibit OGC apoptosis, but the biological mechanism is unclear. The ceRNA mechanism is a prevalent lncRNA mechanism (Smillie et al., 2018). The lncRNA/microRNA (miRNA)/messenger RNA (mRNA) axis significantly contributes to processes such as apoptosis, energy metabolism, and cellular communication (Braga et al., 2020). The overexpression of miR-10b-3p, an emerging microRNA recognized for promoting apoptosis, significantly increased sorafenib-induced apoptosis in hepatocellular carcinoma cells (Shao et al., 2022). Furthermore, miR-10b-3p participates in mammary gland development via circRNA-mediated competitive binding mechanisms (Sahito et al., 2023). However, its role in DOR remains unknown. Forkhead box O 3a (FOXO3a) is a key protein in the FOXO family (Ding et al., 2025), regulated by multiple miRNAs. It plays a crucial role in embryonic development, follicle maturation, and apoptosis (Jia et al., 2014; Liu et al., 2018). Increased levels of phosphorylated FOXO3a in the ovaries of neonatal rats inhibit oocyte apoptosis (Liu et al., 2009). Bioinformatics analysis has identified a potential interaction between NEAT1, miR-10b-3p, and FOXO3a, suggesting a regulatory axis that may play a role in DOR.

Based on this hypothesis, we designed experiments to verify the role of this regulatory axis in OGCs. First, the function of miR-10b-3p was assessed by manipulating its expression in the KGN cell line, using both overexpression and repression techniques. Additionally, dual luciferase reporter gene assay was employed to confirm the regulatory relationships between NEAT1, miR-10b-3p, and FOXO3a. Additionally, qRT-PCR was employed to assess the expression levels of NEAT1, miR-10b-3p, and FOXO3a in OGCs of both EZTGF group, Placebo group, and patients with NOR. This study aimed to verify the clinical efficacy of EZTGF for DOR patients and to explore the molecular mechanisms through which EZTGF enhances IVF-ET treatment outcomes in patients with DOR.

## 2 Methods

### 2.1 Research protocol

**FIGURE 1:**
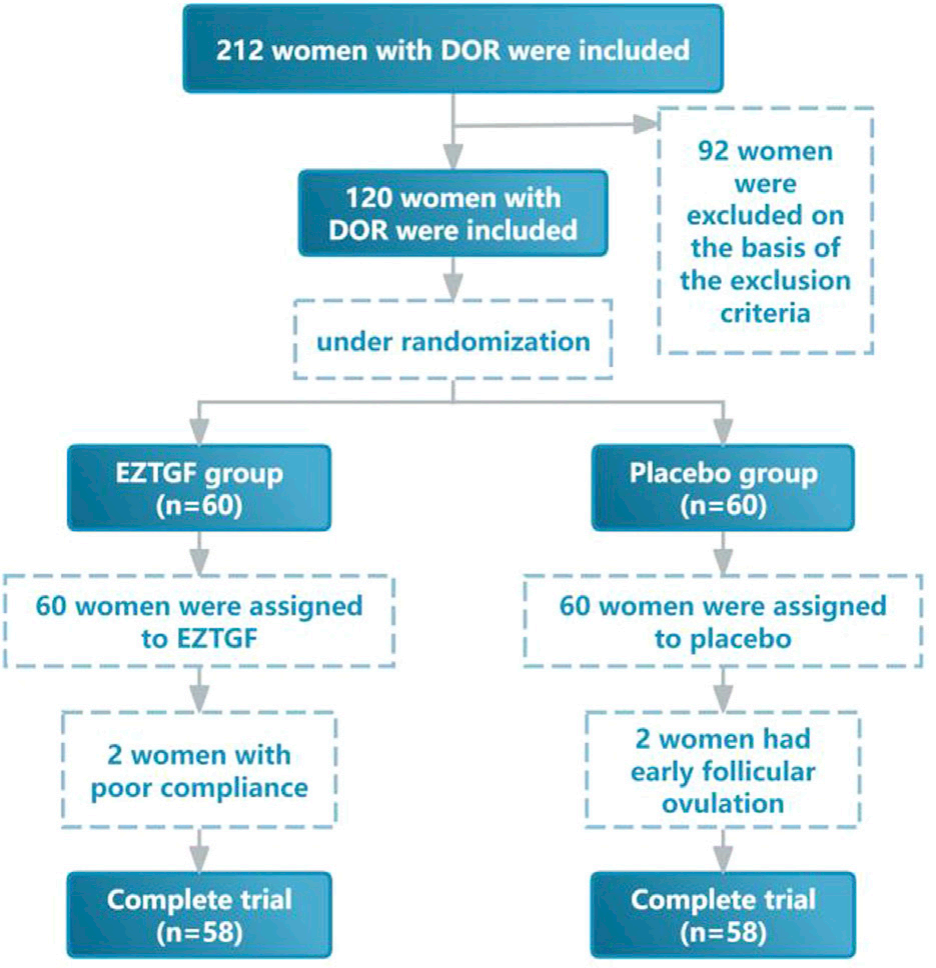
Flowchart of clinical trial of EZTGF in IVF-ET patients with DOR.

This trial was registered in the China Clinical Trial Registry (ChiCTR2100052522). From March 2021 to December 2021, a total of 120 DOR participants undergoing IVF-ET were recruited for this study at the Reproduction and Genetics Centre of the Affiliated Hospital of Shandong University of TCM. A randomized controlled Placebo trial was conducted by random number table to allocate study participants in a 1:1 ratio. Specifically, DOR patients were randomly allocated to the EZTGF group (n = 60), receiving the herbal intervention, or the Placebo group (n = 60), receiving a Placebo as a control. The effect of EZTGF on pregnancy outcomes in DOR was then analyzed. To further investigate the molecular changes in OGCs in DOR and the mechanism of action of EZTGF, an additional 60 patients with NOR were recruited. OGCs were collected from these patients on the day of oocyte retrieval, and the expression of NEAT1, miR-10b-3p, and FOXO3a was compared across groups. During treatment, two patients in the EZTGF group withdrew (due to poor compliance), as did two patients in the Placebo group (due to premature follicular ovulation); a flowchart of the study design is shown in Figure 1. Both herbal and Placebo interventions were initiated on day 2 or 3 of the menstrual cycle prior to ovarian stimulation and continued until discontinuation on the day of triggering. Informed consent was obtained from all study participants.

### 2.2 Inclusion criteria

EZTGF group and Placebo group patients must meet two or more of the following criteria: 1) serum anti-Müllerian hormone (AMH) < 0.5–1.1 ng/mL; 2) an antral follicle count (AFC) < 5–7; and 3) 25 IU/L ≥ a basal FSH level ≥10 IU/L in two consecutive menstrual cycles.

### 2.3 Exclusion criteria

1) age >42 years; 2) BMI ≥35 (kg/m^2^) 3) congenital or acquired abnormalities of the uterine anatomy; 4) chromosomal abnormalities; 5) endometriosis, polycystic ovary syndrome, intrauterine adhesions, endometrial polyps, and other reproductive endocrine disorders; 6) abnormalities in heart, liver, and kidney function or accompanying diseases such as serious psychiatric problems; 7) allergy to the test drug; and 8) prior treatment with TCM or hormonal drugs within the past 3 months. Patients meeting one or more of these criteria will be excluded.

### 2.4 Sample size calculation

The primary outcome for estimation was number of oocytes retrieved, with an expected increase of 1 oocyte following the herbal intervention considered significant. With a standard deviation (σ) of 1.5, an alpha (α) of 0.05, and a test power (1 - β) of 0.90, PASS 15.0 calculates that the sample size for each group is 49. To allow for a 10% dropout rate, we enrolled 120 patients with DOR.

### 2.5 EZTGF or Placebo

EZTGF granules are composed of Cuscuta chinensis Lam [Convolvulaceae; Chinese Dodder Seed], Ligustrum lucidum W.T.Aiton [Oleaceae; Fruits of Ligustrum lucidum], Eclipta prostrata (L.) L [Asteraceae; Dried aerial parts of Eclipta prostrata], Lycium barbarum L [Solanaceae; Goji Berry or Wolfberry], Angelica sinensis (Oliv.) Diels [Apiaceae; Chinese Angelica root], Rehmannia glutinosa (Gaertn.) DC [Orobanchaceae; Prepared Rehmannia Root], Ligusticum chuanxiong Hort [Apiaceae; Rhizome of Ligusticum chuanxiong], Paeonia lactiflora Pall [Paeoniaceae; Roots of Paeonia lactiflora Pallas], Cyperus rotundus L [Cyperaceae; vinegar-processed cyperi rhizoma] and Glycyrrhiza uralensis Fisch. ex DC [Fabaceae; Roots of honey-fired licorice]. The granules are packaged in 3 g sachets, with 12 sachets per box. The Placebo is indistinguishable from EZTGF granules in terms of appearance, color, and taste. It is primarily composed of soybean flour, starch, carbon black pigment, and honey, which mimic the physical characteristics of EZTGF granules but contain no active ingredients, thereby ensuring no therapeutic effect. The Placebo is packaged identically to EZTGF granules in terms of daily dosage. This design serves two purposes: first, it prevents participants from identifying their assignment to the Placebo group, which could otherwise compromise their compliance; second, it eliminates the potential confounding effects associated with using vitamins as a Placebo. Participants in the Placebo group will receive the same dosage regimen as those in the EZTGF granule group.

### 2.6 IVF-ET procedure

On days 2–3 of menstruation, the participants underwent assessments for baseline endocrine levels and ultrasound. The physician then established the initial FSH dose according to the test outcomes, as well as considering factors such as age, BMI, AFC, and earlier ovarian responses. All participants were administered recombinant follicle-stimulating hormone (r-FSH), generally within the dosage range of 150–225 U/day. The dosage of r-FSH was modified every 2 days based on the levels of hormones and the follicular growth noted through ultrasound. From day 5 or 6 of ovarian stimulation up until the trigger day, additional treatment with cetrorelix acetate was provided to all participants. Ultimately, once the average diameter of two or more dominant follicles attained 18 mm, the participants were administered a trigger injection of 250 μg recombinant human chorionic gonadotropin. Oocyte retrieval via transvaginal ultrasound-guided ovarian aspiration was conducted 35–37 h following the trigger. The practice of oocyte pick up is based on guidelines issued by the European Society of Human Reproduction and Embryology (ESHRE) (D'Angelo et al., 2019; Gordon et al., 2022). The procedure was performed with a 14-16G puncture needle under the guidance of transvaginal ultrasound. To preserve the integrity of the cumulus-oocyte complex, a low-pressure suction system should be employed, with the pressure strictly maintained below 150 mmHg. During the puncture procedure, the following operational protocols must be adhered to: Push the puncture needle through the needle guide to the top of the vagina, gently puncture the vaginal wall to the bottom of the ovary, and then puncture the nearest follicle in one go. Negative pressure should be initiated after the needle penetrates the ovarian cortex but before entering the target follicle to prevent follicular fluid loss. Upon completion of follicular fluid aspiration, the negative pressure should be released promptly as the needle is withdrawn from the ovarian parenchyma and vaginal wall. This step is critical to minimize shear stress caused by abrupt pressure changes within the tubing system, thereby preventing mechanical damage to the oocytes.

All participants received embryo transfer at either the cleavage stage on day 3 or the blastocyst stage on day 5 post-retrieval. Transfers were canceled if tubal fluid was present, serum progesterone (P) > 1.5 ng/mL before the trigger day, or endometrial transformation day. No more than 2 embryos were transferred at a time.

### 2.7 Luteal support

Progesterone vaginal extended-release gel (90 mg/day) or progesterone (40 mg/day) was used in combination with oral dextroprogesterone tablets (30 mg/day), which were discontinued from the time of embryo transfer to the 10th week of pregnancy. The medication was stopped immediately in case of negative serum β-hCG, biochemical pregnancy, or transvaginal ultrasound suggesting embryonic arrest.

### 2.8 Randomization and blinding

After enrolling patients who volunteered to participate in the study, we generated random numbers and performed block randomization according to R language version 3.5.1, with a 1:1 ratio of participants between the two groups. The EZTGF and Placebo groups were coded with corresponding one-to-one subject codes. The exact details of the groupings were managed by the Centre for Medicines Management. The study medications and Placebos were prepared to resemble each other in appearance and odor. The attending physicians, outcome assessors, statistical analysts, and patients were blinded to the study.

### 2.9 Collection of OGCs

On the day when oocytes were collected, the follicular fluid was acquired from patients receiving IVF and was processed without delay to avoid degradation of RNA and proteins. In summary, OGCs were separated from the follicular fluid through a density gradient technique. The follicular fluid underwent centrifugation at 1,500 rpm for a duration of 15 min at room temperature. After this process, the supernatant was removed, and the cellular pellet was resuspended in phosphate-buffered saline (PBS) that had been pre-warmed. The human peripheral blood lymphocyte isolation solution was carefully layered with the cellular suspension in a 1:1 ratio and subsequently centrifuged at 2,000 rpm for 20 min at ambient temperature. Following centrifugation, the contents of the tube formed three distinct layers, and the white misty layer in the middle was carefully aspirated into a new centrifuge tube, which contained the isolated GCs. These cells were immediately frozen and stored at −80°C for further analysis.

### 2.10 Cell culture and transfection

Experiments were performed using the KGN cell line, an immortalized cell line commonly used to study the function of OGCs (Nishi et al., 2001). KGN cells were commonly grown in DMEM/F-12 medium enriched with 10% fetal bovine serum, in addition to 100 U/mL of penicillin and 100 U/mL of streptomycin. The cultures were kept at 37°C within an environment containing 5% CO_2_ and 95% humidity. When the cell density reached 60%–80%, OE-NEAT1, si-NEAT1, mimic-miR-10b-3p, inhibitor-miR-10b-3p, and the corresponding negative control were transfected into KGN cells. Functional assays were performed after 24–48 h.

### 2.11 EDU experiment

In summary, KGN cells were seeded into 96-well plates and allowed to culture until they attained standard growth levels. A suitable volume of 50 μM EdU medium was then prepared, added to the wells, and incubated for less than 2 h. Following this, 100 μL of 50 μM EdU medium was introduced to every well and incubated for 2 h before the medium was removed. After the cells were fixed, they underwent staining with fluorescent dyes, and images of the stained cells were obtained with a fluorescence microscope.

### 2.12 Flow cytometry

Cells were treated with trypsin and subsequently incubated for 30 min. They were then washed two times using pre-cooled PBS and centrifuged at 300 g for 5 min at 4°C. Around 1–5 × 10^5^ cells were collected, resuspended in PBS, and placed on ice at room temperature, shielded from light for 10–15 min. For cell resuspension, either 400 μL of PBS or 1× binding buffer was added to each tube, followed by analysis on the machine.

### 2.13 Data analyses

Data that follow a normal distribution are presented as mean ± standard deviation (x̄ ± s), while data that do not follow a normal distribution are summarized using median and interquartile range. Numerical data are reported as frequencies and percentages (%). If the data from both groups met the requirements for normal distribution, an independent samples t-test was applied; if not, non-parametric tests were used. The chi-square test was utilized for categorical data comparisons, while Fisher’s exact test was applied when the sample size fell below 40 or when over 20% of expected frequencies were below 5.

## 3 Results

### 3.1 Baseline characteristics of participants

No notable differences were observed in the baseline characteristics of the two groups, such as the ages of females and males, duration of infertility, types of infertility, BMI, basal endocrine levels, AFC, number of embryos transferred, quantity of high-quality embryos transferred, and endometrial thickness (Table 1, P > 0.05).

**TABLE 1:** Baseline characteristics in participants.

	EZTGF group (n = 58)	Placebo group (n = 58)	P
Age (years)	35 (33–37)	35 (33–36)	0.555
Male age (years)	34 (32.75–37)	35 (33–36)	0.925
Type of infertility			0.697
Primary infertility	36.2% (21/58)	32.8% (19/58)	
Secondary infertility	63.8% (37/58)	67.2% (39/58)	
Infertility duration (years)	2 (1–4)	3 (2–5)	0.37
BMI (kg/m^2^)	22.6 (20.2–24.13)	23 (21.5–24.13)	0.281
Basal FSH level (IU/L)	10.32 (7.9–12.1)	9.53 (7.05–11.15)	0.209
Basal LH level (IU/L)	4.8131 ± 1.69	4.5241 ± 2.26	0.437
Basal E2 level (pg/mL)	39.7336 ± 13.49	40.031 ± 17.95	0.92
Basal P4 level (ng/mL)	0.46 (0.33–0.58)	0.41 (0.25–0.61)	0.438
Basal T level (ng/mL)	0.38 (0.21–0.49)	0.35 (0.29–0.41)	0.673
AMH (ng/mL)	1 (0.67–1.1)	0.89 (0.64–1.26)	0.114
AFC (No.)	4 (3–6)	3.5 (3–5)	0.07
Embryos transferred (No.)			0.346
1	37.04% (20/54)	37.74% (20/53)	
2	62.96% (34/54)	62.26% (33/53)	
High-quality embryos transferred (No.)			0.643
0	48.15% (26/54)	52.83% (28/53)	
1	38.89% (21/54)	39.62% (21/53)	
2	12.96% (7/54)	7.55% (4/53)	
Thickness of endometrium (mm)	10 (9–11.5)	10.75 (9–12)	0.479

### 3.2 IVF-related outcomes

In the EZTGF group, the count and proportion of high-quality embryos were considerably greater compared to the Placebo group (Table 2, P < 0.05). It is important to note that there was a significant rise in the number of high-quality embryos at the cleavage stage within the EZTGF group (Table 2, P < 0.05).

**TABLE 2:** IVF-related outcomes in participants.

	EZTGF group (n = 58)	Placebo group (n = 58)	P
E_2_ (pg/mL)	721.05 (587.3–1049.38)	713.06 (560.87–1098.43)	0.899
P_4_ (ng/mL)	0.96 (0.76–1.21)	1.01 (0.75–1.12)	0.412
Oocytes acquired (No.)	3 (3–5)	3 (2–4)	0.164
Embryo (No.)	2 (2–3)	2 (2–3)	0.161
Cleavage stage embryos (No.)	2 (2–2)	2 (2–2)	0.347
Blastocysts (No.)	0 (0–1)	0 (0–1)	0.226
High-quality embryos (No.)	1 (0–2)	1 (0–1)	**0.007**
High-quality cleavage stage embryos (No.)	1 (0–1)	0.5 (0–1)	**0.024**
High-quality blastocysts (No.)	0 (0–0)	0 (0–0)	0.089
Normal fertilisation rate	81.38% (153/188)	80.39% (123/153)	0.054
Blastocyst formation rate	61.70% (29/47)	65.38% (17/26)	0.755
High-quality embryo rate	44.36% (59/133)	31.90% (37/116)	**0.044**

Bold values indicate a significant difference of less than 0.05.

No meaningful differences were detected between the two groups regarding the count of eggs retrieved, levels of E2 and P on the trigger day, number of available embryos, count of cleavage stage embryos, total blastocyst numbers, or the quantity of high-quality blastocysts. Furthermore, the two groups exhibited no significant variations in normal fertilization rates or rates of blastocyst formation.

### 3.3 Pregnancy outcomes

No significant statistical differences were found between the two groups regarding clinical pregnancy, biochemical pregnancy, ectopic pregnancy, miscarriage, or live birth rates (Table 3, P > 0.05).

**TABLE 3:** Outcome of pregnancy in participants.

	EZTGF group (n = 58)	Placebo group (n = 58)	P
Clinical pregnancy	42.59% (23/54)	33.96% (18/53)	0.359
Biochemical pregnancy	44.44% (24/54)	35.85% (19/53)	0.365
Ectopic pregnancy	4.35% (1/23)	0.00% (0/18)	>0.999
Pregnancy loss	17.39% (4/23)	33.33% (6/18)	0.289
Live birth	33.33% (18/54)	22.64% (12/53)	0.218

### 3.4 Adverse reactions

No adverse reactions were reported to the course of treatment in any patients.

### 3.5 miR-10b-3p inhibits proliferation and promotes apoptosis in GCs

**FIGURE 2:**
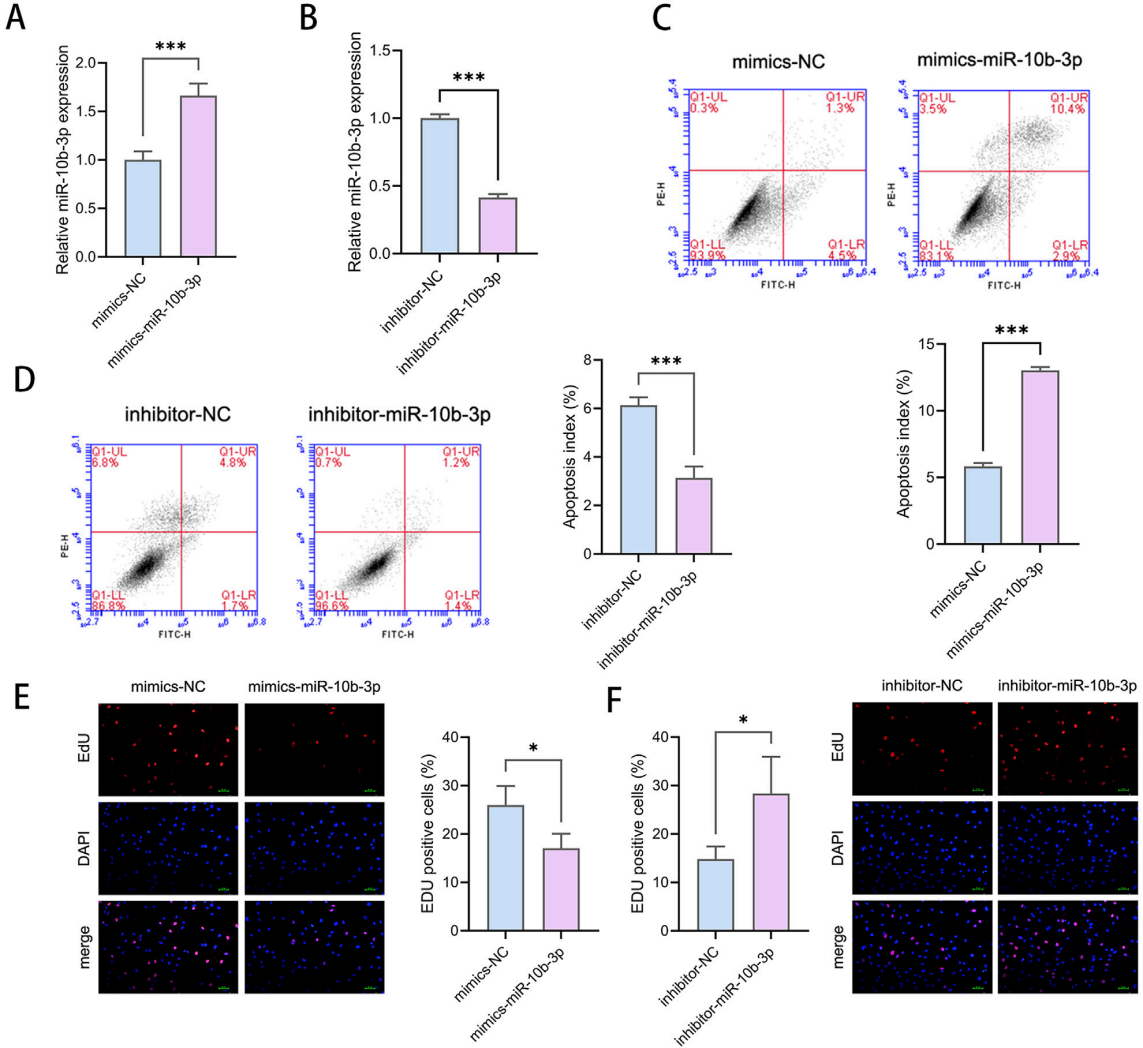
miR-10b-3p inhibits proliferation and promotes apoptosis in GCs **(A, B)** qRT-PCR showing overexpression of miR-10b-3p as well as inhibition of miR-10b-3p efficiency. **(C, D)** Flow cytometry to detect the proportion of apoptotic cells. **(E, F)** EDU assay to detect cell proliferation ability. Notes: *P < 0.05; **P < 0.01; ***P < 0.001.

We performed transfection of KGN cells with mimic-miR-10b-3p, inhibitor-miR-10b-3p, and their respective negative controls, and confirmed the transfection efficiency using qRT-PCR (Figures 2A, B). An increased expression of miR-10b-3p significantly reduced the proliferative ability of KGN cells (Figure 2E), decreased the proportion of viable cells, and increased the proportion of dead cells (Figure 2C). Conversely, transfection with inhibitor-miR-10b-3p resulted in significant enhancement of KGN cell proliferation (Figure 2F) along with an increase in the proportion of live cells and decrease in the proportion of dead cells (Figure 2D). These findings suggest that miR-10b-3p promotes apoptosis and inhibits the proliferation of GCs.

### 3.6 NEAT1 directly targets miR-10b-3p to promote cell proliferation and inhibit apoptosis in GCs

**FIGURE 3:**
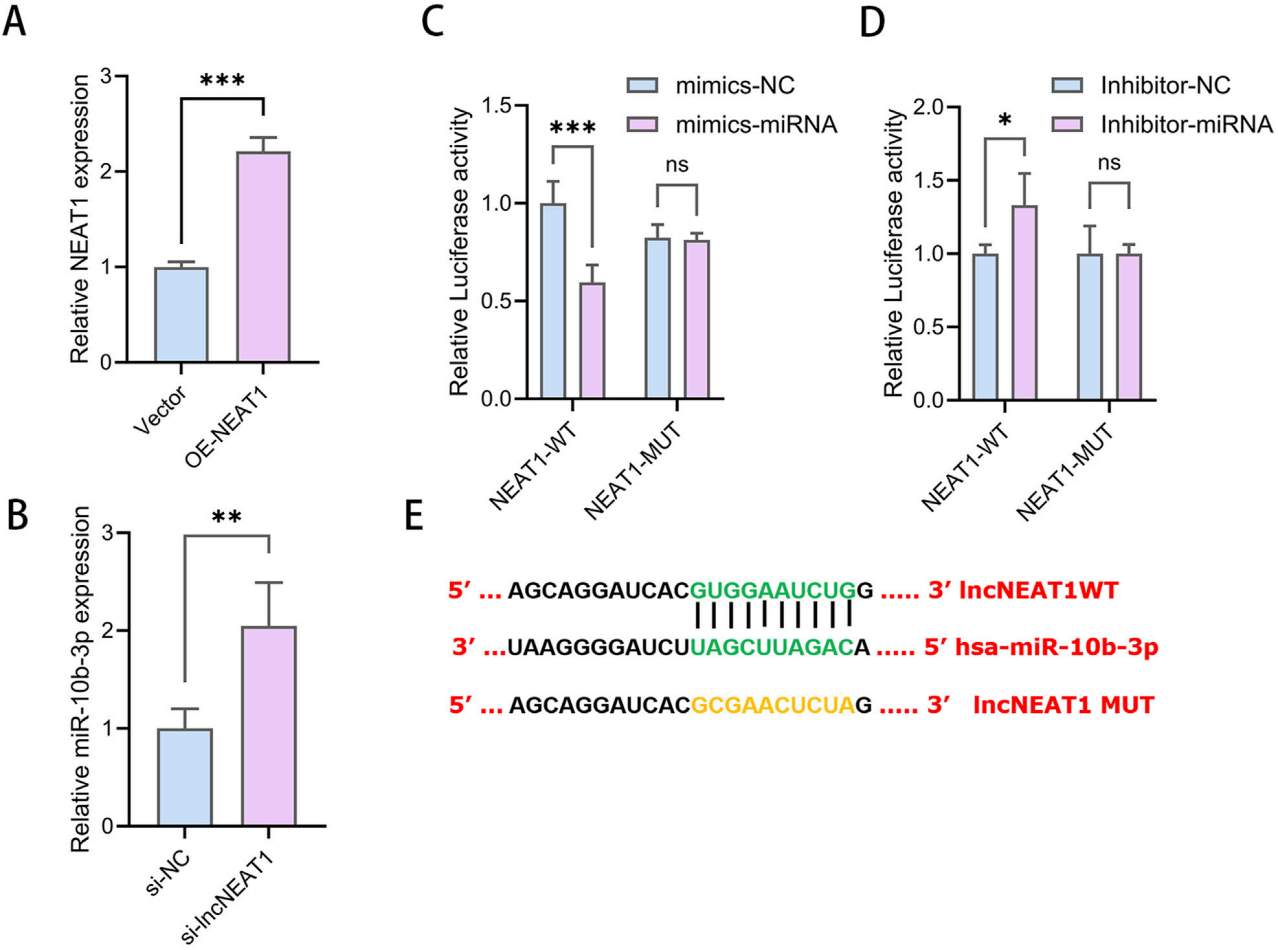
NEAT1 directly targets miR-10b-3p to promote cell proliferation and inhibit apoptosis in GCs **(A, B)** qRT-PCR to detect the relative expression of miR-10b-3p after overexpression and knockdown of NEAT1. **(C–E)** Dual luciferase reporter gene assay to detect the targeting relationship between NEAT1 and miR-10b-3p. Notes: *P < 0.05; **P < 0.01; ***P < 0.001.

The overexpression of NEAT1 significantly reduced the relative expression of miR-10b-3p in KGN cells (Figure 3A), while NEAT1 inhibition increased miR-10b-3p expression (Figure 3B), suggesting a negative regulatory relationship between NEAT1 and miR-10b-3p. Bioinformatics analyses revealed potential binding sites between these molecules (Figure 3E). To verify direct binding, dual luciferase reporter gene assays were performed. We first constructed wild-type and mutant sequences of NEAT1. KGN cells were then co-transfected with either mimic-NC or mimic-miR-10a-3p alongside these sequences. The overexpression of miR-10b-3p significantly decreased luciferase activity at the NEAT1-WT binding site, with no effect on NEAT1-MUT in KGN cells (Figure 3C). Similarly, inhibition of miR-10b-3p significantly increased luciferase activity at the NEAT1-WT binding site but had no effect on NEAT1-MUT (Figure 3D). These findings suggest that NEAT1 can directly target the 3′UTR region of miR-10b-3p and plays an important role in the function of GCs.

### 3.7 Direct targeting of FOXO3a by miR-10b-3p inhibits cell proliferation and promotes apoptosis in GCs

**FIGURE 4:**
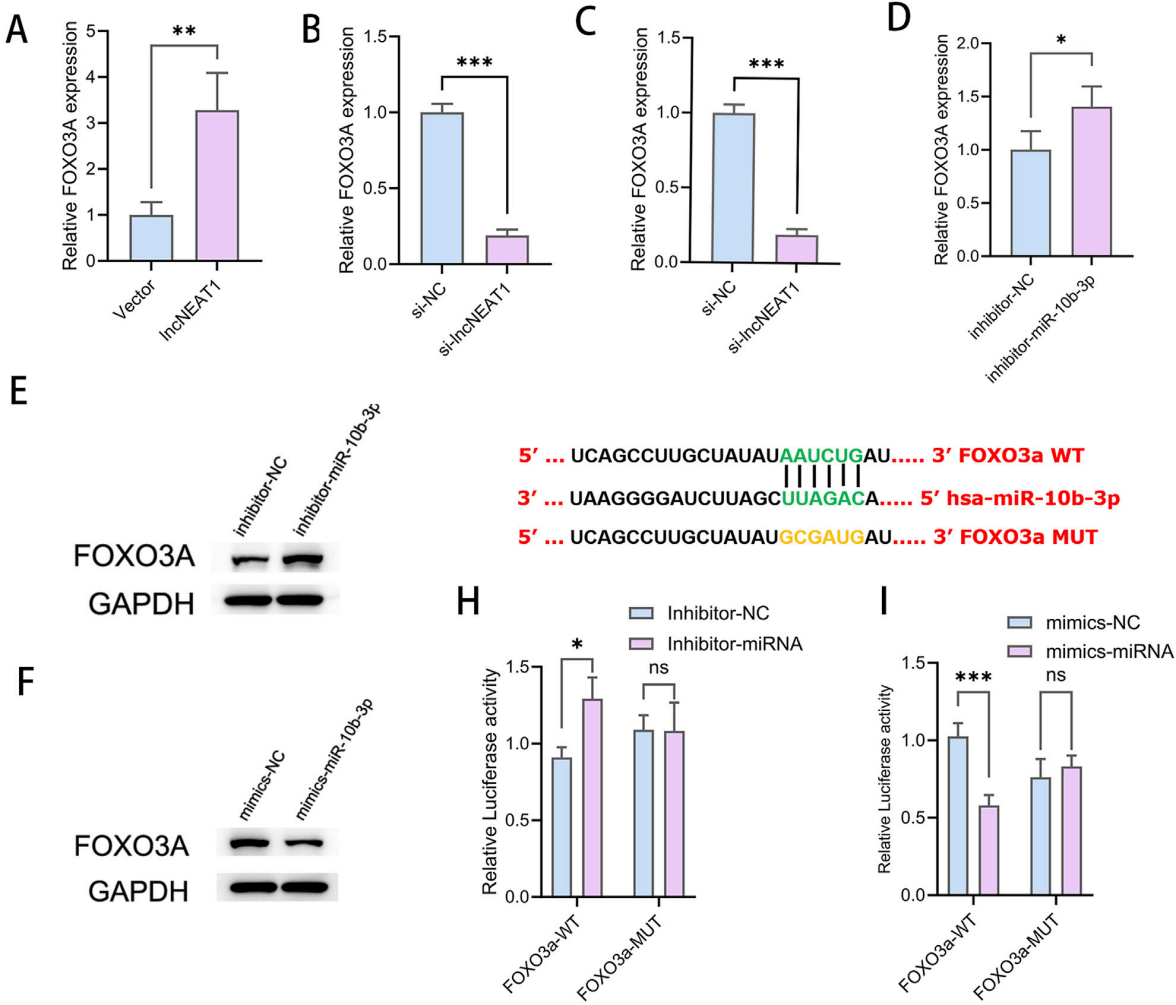
Direct targeting of FOXO3a by miR-10b-3p inhibits cell proliferation and promotes apoptosis in GCs **(A, B)** qRT-PCR to detect the relative expression of FOXO3a after overexpression and knockdown of NEAT1. **(C, D)** Relative expression of FOXO3a after qRT-PCR detection of overexpression and knockdown of miR-10b-3p. **(E, F)** WB detection of relative FOXO3a protein expression after overexpression and inhibition of miR-10b-3p. **(G–I)** Dual luciferase reporter gene assay to detect the targeting relationship between miR-10b-3p and FOXO3a.Notes: *P < 0.05; **P < 0.01; ***P < 0.001.

Overexpression of NEAT1 led to a significant increase in the mRNA expression of FOXO3a (Figure 4A), while silencing NEAT1 decreased FOXO3a levels (Figure 4B), indicating a positive regulatory relationship between NEAT1 and FOXO3a. Conversely, miR-10b-3p overexpression resulted in a reduction of FOXO3a expression at both the transcript and protein levels (Figures 4C,F). Inhibition of miR-10b-3p led to increased FOXO3a expression (Figures 4D,E), suggesting a negative regulatory effect of miR-10b-3p on FOXO3a. It is well known that miRNAs typically function by targeting the 3′-UTR region of mRNA to inhibit translation and destabilize the transcript. Biosignature analysis revealed potential binding sequences between miR-10b-3p and FOXO3a (Figure 4G). To confirm the direct targeting relationship between miR-10b-3p and FOXO3a, we conducted dual luciferase reporter gene experiments (Figures 4H,I). Our results suggest that miR-10b-3p directly targets FOXO3a, playing a crucial role in inhibiting cell proliferation and promoting apoptosis in GCs.

### 3.8 EZTGF partially reversed the low expression of NEAT1 and FOXO3a expression and the high expression of miR-10b-3p in OGCs from DOR patients

**FIGURE 5:**
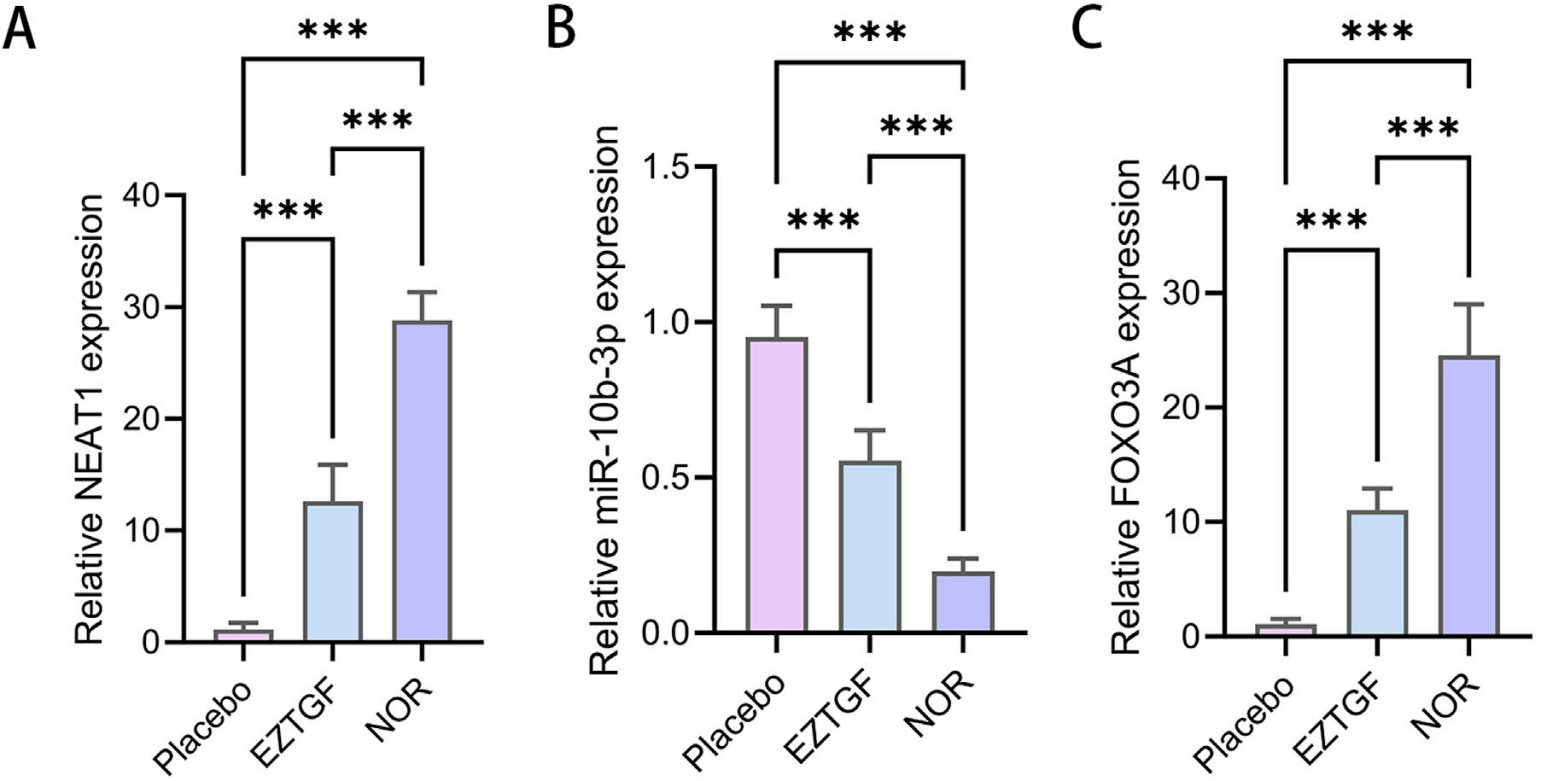
EZTGF partially reversed the low expression of NEAT1 and FOXO3a expression and the high expression of miR-10b-3p in OGCs from DOR patients **(A–C)** Relative expression of NEAT1, miR-10b-3p, and FOXO3a in female ovarian granulosa cells in the DOR group (Placebo group vs. diastereodecane group) as well as in the NOR group. Notes: ***P < 0.001. nuclear paraspeckle assembly transcript 1, NEAT1; Forkhead box O3a, FOXO3a.

NEAT1 and FOXO3a expression were significantly downregulated in the OGCs from DOR patients compared to NOR patients (Figures 5A,C), while miR-10b-3p expression was significantly upregulated (Figure 5B). Notably, in the EZTGF group, NEAT1 and FOXO3a expression levels were significantly higher (Figures 5A,C), and miR-10b-3p expression was significantly lower (Figure 5B) compared to the Placebo group.

#### 3.8.1 Permission to reuse and copyright

Our histogram (Figures 1–5) used in the resulting graph was independently created by our team members based on the experimental data collected in this study. The histograms were drawn using the software GraphPad Prism 10, for which we have obtained proper authorization. Attached is the Copyright Authorization Certificate from GraphPad Prism 10. Additionally, the data used in the figures are original and generated solely by our experiments, ensuring there are no third-party copyright implications. Moreover, graphical abstract was created by our team members using the Figdraw platform, for which we have obtained authorization.

## 4 Discussion

The etiology of DOR is very complex and associated with genetics, age, and surgical injury; however, its pathogenesis is unclear (Chen et al., 2024). Patients may experience normal or abnormal menstruation, and by the time a distinct phenotype becomes apparent, ovarian function is often severely compromised. IVF is a valuable tool for addressing infertility in patients with DOR; however, pregnancy rates remain low. This is largely due to the reduced number of oocytes and poorer oocyte quality in these patients. Therefore, developing new therapies to improve the number and quality of oocytes in DOR is necessary. Oocyte development is synergistically regulated by GCs (Gioacchini et al., 2018). Excessive apoptosis of GCs is a key cause of decreased oocyte quality. When GC apoptosis reaches >10%, the oocytes are rapidly depleted, and follicular atresia occurs, resulting in DOR (Bulgurcuoglu Kuran et al., 2022; Fan et al., 2019). According to TCM, the kidney is a subtle organ that promotes growth and development and maintains the normal reproductive function of the human body. Insufficient yin essence in the kidneys results in a lack of biochemical sources of GCs, which hinders oocyte development.

EZTGF functions as a tonic for the kidney and yin and promotes reproduction. It is based on the TCM theory and a special combination of Chinese medicines and has a multi-pathway and multi-targeted mechanism of action. EZTGF consists of Er Zhi Wan and Si Wu Tang Jia Wei. The formulation includes Cuscuta chinensis Lam, Ligustrum lucidum W.T.Aiton, Eclipta prostrata (L.) L, Lycium barbarum, Rehmannia glutinosa (Gaertn.) DC, Cyperus rotundus L, Glycyrrhiza uralensis Fisch. ex DC and so on. This formula contains Cuscuta chinensis Lam and Ligustrum lucidum W.T.Aiton as the main herbs. Cuscuta chinensis is pungent, sweet, and slightly warm, and enters the liver and kidney meridians, which can tonify the liver and kidney and benefit the essence. Total flavonoids from Cuscuta chinensis act as γ-secretase agonists and exert a protective effect on ovarian germline stem cells through the Notch signaling pathway (Li et al., 2024). Ligustrum lucidum have a sweet and bitter flavor and are flat in nature, tonifying the essence of kidney yin. Oleanolic acid is the active ingredient in Ligustrum lucidum, which has significant anti-inflammatory and anti-aging activities. Oleanoic acid can effectively restore testicular function by inhibiting the activation of the NF-κB, p53, and p38 signaling pathways and attenuating DNA damage and apoptosis in germ cells (Zhao et al., 2017). The combination of Rehmannia glutinosa and Eclipta prostrata helps replenish kidney essence, nourish the bone marrow, and tonify liver and kidney yin, offering benefits for yin and blood. Lycium barbarum is sweet and enters the liver and kidney meridians to nourish yins and moisten dryness. Cyperus rotundus is used to open depressions, promote Qi circulation, and enter the liver meridian. Licorice acts as an enabler, enhancing the overall effects of the medicine combination, which nourishes the kidney and blood and regulates Chong Ren. In this randomized, double-blind, placebo-controlled EZTGF trial, the efficacy of EZTGF and Placebo was observed. Our results showed that the rate of high-quality embryos in patients with DOR treated with EZTGF was significantly higher than that in the control group, accompanied by a notable increase in the number of high-quality embryos. Improved embryo quality in DOR patients is associated with higher implantation potential and developmental competence. The infertility factors in DOR are multifaceted, with some patients presenting with concurrent conditions such as tubal obstruction, poor endometrial receptivity, or insufficient endometrial thickness. These individual variations may explain the occurrence of ectopic pregnancies or biochemical pregnancies in both the EZTGF and Placebo groups, highlighting the complexity of DOR management.

According to TCM theory and the above studies, EZTGF can improve the reproductive function of patients with DOR through multiple mechanisms. We further found that EZTGF may inhibit apoptosis of granulosa cells by targeting the NEAT1/miR-10b-3p/FOXO3a pathway. In this study, the expression levels of lncNEAT1, miR-10b-3p and FOXO3a in test group, control group and blank control group were detected by RT-qPCR. After treatment with TCM, levels of lncNEAT1 expression in GCs of patients with DOR was significantly upregulated. In addition, the expression level of miR-10b-3p was significantly downregulated, and the expression level of FOXO3a was significantly upregulated. NEAT1 is a long non-coding RNA, identified in our previous studies as a key RNA that is significantly underexpressed in DOR, and reportedly regulates apoptosis through multiple mechanisms (Ding et al., 2017; Liu et al., 2020; Liu et al., 2019). A key mRNA molecule in non-obstructive azoospermia, miR-10b-3p is significantly over-expressed in testicular tissue (Zhang H. T. et al., 2020). Recent studies demonstrate that paternal immune activation specifically regulates miR-10b-3p expression through sperm epigenetic reprogramming, mediating transgenerational neurobehavioral abnormalities (Kleeman et al., 2024). In oncodiagnostics, miR-10b-3p has been identified as a biomarker capable of distinguishing pancreatic paragangliomas (PGL) from pancreatic neuroendocrine tumors (PanNETs) (Enguita et al., 2023). Notably, miR-10b-3p can inhibit the expression of FOXO3 protein by targeting the 3′-untranslated region, which promotes cell proliferation, colony formation, and migration and invasion of esophageal squamous cell carcinoma (Lu et al., 2018). These effects ultimately promote the development of hepatocellular carcinoma. They found that when the phosphorylation of FOXO3a was reduced, the relative expressions of caspase-3 and rates of cell apoptosis increased (Zhang et al., 2020b). This research shows that activation of FOXO3a phosphorylation can inhibit apoptosis and improve ovarian function. Experiments on KGN cells showed that lncNEAT1 inhibited apoptosis of GCs and promoted their proliferation. Conversely, miR-10b-3p promoted apoptosis and inhibited proliferation of GCs. Combined with the above experimental results, we believe that EZTGF can upregulate the expression of NEAT1, downregulate miR-10b-3p, and promote the phosphorylation of FOXO3a, thereby reducing the apoptosis rate of GCs. These effects will improve oocyte quality and embryonic development potential, and will ultimately improve the high-quality embryo rate of patients with DOR who undergo IVF-ET.

TCM therapies, including herbal formulations and acupuncture, have been demonstrated to improve hormonal levels and increase AFC in patients with DOR (Lin et al., 2023; Wang et al., 2016; Zhou et al., 2022). However, the majority of clinical trials have focused solely on observing changes in hormonal levels and ultrasound indicators, with limited follow-up periods, thus failing to comprehensively evaluate their long-term impact on reproductive outcomes. This study focuses on DOR patients undergoing IVF-ET, with the number of oocytes retrieved as the primary efficacy indicator. By directly assessing oocyte and embryo quality and extending follow-up to pregnancy outcomes, this study provides a more comprehensive evaluation system for therapeutic efficacy. In terms of pharmacological mechanism research, this study combines clinical trials with cellular experiments to explore the clinical efficacy and molecular mechanisms at multiple levels. Not only does it validate the significant improvement in embryo quality in DOR patients treated with EZTGF, but it also, for the first time, reveals its molecular mechanism of inhibiting OGC apoptosis through the regulation of the NEAT1/miR-10b-3p/FOXO3a signaling pathway. This provides new theoretical foundations and research directions for the application of TCM in the field of assisted reproduction. This discovery not only enriches the theoretical understanding of TCM mechanisms in treating DOR, but also lays a scientific foundation for developing precision treatment plans based on TCM formulations.

The results of this study demonstrate that EZTGF significantly improves the quantity and proportion of high-quality embryos in patients with DOR, particularly highlighting its efficacy in enhancing cleavage-stage embryos. This finding provides robust support for the application of EZTGF in improving IVF-ET outcomes for DOR patients. EZTGF can serve as an adjunctive treatment option for DOR patients, especially in cases where traditional ovulation induction protocols yield suboptimal results, offering a novel approach to enhance treatment efficacy. Given its inhibitory effect on OGC apoptosis, EZTGF may also be integrated into long-term management strategies for DOR patients with fertility aspirations, potentially delaying further decline in ovarian reserve by suppressing OGC apoptosis and improving ovarian function. Furthermore, the expression levels of NEAT1, miR-10b-3p, and FOXO3a may serve as potential biomarkers for assessing ovarian function and treatment.

This randomized, double-blind, placebo-controlled trial demonstrated that EZTGF treatment significantly improved the high-quality embryo rate and the number of top-grade embryos in patients with DOR. However, this study had some limitations. As a preliminary exploratory study, the limited sample size and study duration did not allow for a comprehensive comparison between EZTGF and existing marketed formulations. Future studies incorporating clinical trials comparing EZTGF with other TCM therapies for DOR patients are warranted, as this would provide further validation of EZTGF’s efficacy. In addition, this was a single-centre study with a sample source limited to Shandong, China. Furthermore, the pharmacological mechanism of EZTGF is still not well-studied. Research on the drug-containing serum and the effective blood components of EZTGF may be the next step and should be conducted at both the cellular and organismal levels. Therefore, follow-up clinical trials and basic complementary studies are still necessary.

Overall, our study showed that EZTGF significantly increased the rate of high-quality embryos in DOR patients who undergo IVF-ET, and the mechanism of treatment may be achieved by inhibiting GC apoptosis through expression of NEAT1/miR-10b-3p/FOXO3a.

## Data Availability

Data will be made available on request. Requests to access the datasets should be directed to the corresponding authors.
